# *PvPR10-3* Expression Confers Salt Stress Tolerance in *Arabidopsis* and Interferes with Jasmonic Acid and ABA Signaling

**DOI:** 10.3390/plants14193092

**Published:** 2025-10-07

**Authors:** Kaouthar Feki, Hanen Kamoun, Amal Ben Romdhane, Sana Tounsi, Wissal Harrabi, Sirine Salhi, Haythem Mhadhbi, Maurizio Trovato, Faiçal Brini

**Affiliations:** 1 Biotechnology and Plant Improvement Laboratory, Centre of Biotechnology of Sfax, Sfax 3018, Tunisia; kaouther.feki@cbs.rnrt.tn (K.F.); kamoun.hanene@gmail.com (H.K.); sana.tounsi@esakef.u-jendouba.tn (S.T.); harrabiwissal312@gmail.com (W.H.); salhisirine135@gmail.com (S.S.); 2 Research Laboratory of Environment Toxicology-Microbiology and Health, Faculty of Science of Sfax, University of Sfax, Sfax 3000, Tunisia; amal.ben.romdhane@gmail.com;; 3 Higher School of Agriculture of Kef (ESAK) Boulifa Campus, University of Jandouba, Kef 7100, Tunisia; 4 Laboratory of Legumes and Sustainable Agrosytems (L2AD), Centre of Biotechnology of Borj-Cedria (CBBC), Hammam-Lif 2050, Tunisia; haythem.mhadhbi@cbbc.rnrt.tn; 5 Department of Biology and Biotechnology, Sapienza University of Rome, 00185 Rome, Italy; maurizio.trovato@uniroma1.it

**Keywords:** combined stress, common bean, phytohormone, pathogenesis-related PR10 protein, salt stress

## Abstract

Salt stress is a major abiotic factor limiting crop productivity worldwide, as it disrupts plant growth, metabolism, and survival. In this study, we report that the genes *PvPR10-2* and *PvPR10-3* were significantly up-regulated in bean leaves and stems in response to combined salt and jasmonic acid (NaCl–JA) treatment. Foliar application of JA with salt induced physiological alterations, including stem growth inhibition, H_2_O_2_ accumulation, and activation of antioxidant enzymes. To investigate the role of *PvPR10-3* in response to salt and phytohormones, we introduced this gene into *Arabidopsis* and found that its heterologous expression conferred salt tolerance to the transgenic lines. Interestingly, exogenous JA contributed to salt tolerance by reducing H_2_O_2_ levels, inducing ROS-scavenging enzymes, and promoting the accumulation of phenolic compounds and ABA. Furthermore, gene expression analysis of the transgenic lines revealed that *PvPR10-3* expression under NaCl–JA stress is associated with the induction of JA-related genes like *MYC2*, *JAZ2*, *JAZ11*, and *JAZ12*, as well as SA-responsive genes, like *ALD1* and *TGA2,* and two ABA-independent components *DREB2A* and *ERD1*, suggesting potential coordination between JA, ABA, and SA signaling in salt stress response. Additionally, key flowering regulators (*FT*, *GI*) were upregulated in transgenic lines under NaCl–JA treatment, suggesting a previously unexplored link between salt tolerance pathways and the regulation of flowering time. Taken together, our findings suggest a role of *PvPR10-3* in enhancing salt stress tolerance and the involvement of exogenous JA in tolerance potentially by modulating ROS balance, hormone-associated gene expression, and protective secondary metabolites.

## 1. Introduction

In their environment, crops face challenging stress conditions like soil salinity, drought, heat waves, and floods that cause a negative impact on agriculture by decreasing yield and threatening food security [1,2]. Salinity is among the major abiotic stresses that significantly reduce crop productivity and quality. Indeed, crops grown in saline soils suffer from osmotic stress, ion imbalance, toxicity, and oxidative stress [3]. When plants are confronted with environmental stresses, cells sense and respond to stress conditions via the activation of specific cellular signals, which coordinate with various processes, like stomatal dynamics, gene expression, and growth, to achieve stress tolerance [4]. The perception of salt stress occurs mainly through excess apoplastic Na^+^, high levels of cytosolic Ca^2+^, and reactive oxygen species (ROS) accumulation [3]. A consequence of this stress perception is a disruption of cellular homeostasis, leading to the overproduction of ROS. In plant cells, a high amount of ROS, especially H_2_O_2_, leads to loss of genomic stability, damage to cell membranes and cellular components, and eventually cell death [5]. To mitigate oxidative damage, plants have evolved a ROS-scavenging system including enzymatic and non-enzymatic components. Key enzymatic antioxidants include catalase (CAT), peroxidase (POD), superoxide dismutase (SOD), peroxiredoxins (PRX), thioredoxin (TRX), and ascorbate-glutathione cycle enzymes like monodehydroascorbate reductase (MDHAR), dehydroascorbate reductase (DHAR), and glutathione reductase (GR). Various reports demonstrate the involvement of these antioxidant enzymes not only in salt stress but also in other abiotic stress [6,7,8,9]. Furthermore, various non-enzymatic molecules like glutathione, ascorbate, phenolic compounds, and flavonoids are involved in maintaining ROS homeostasis [10]. Moreover, to cope with salt stress, plants produce osmolytes such as proline, glycine betaine, and soluble carbohydrates in their cells for osmotic adjustment [11,12]. Intriguingly, a recent model proposes that ROS homeostasis and the efficacy of the enzymatic system are coordinated with the accumulation of the compatible solute proline, which itself can act as a non-enzymatic antioxidant and redox buffer [13]. Under salt stress, the ion balance regulation occurs via the involvement of different transporters, mainly the membrane Na^+^/H^+^ antiporter Salt-Overly-Sensitive 1 (SOS1), which excludes the excess Na^+^ from the cytosol to the apoplast, and the vacuolar Na^+^/H^+^ exchanger (NHX) that sequesters the toxic Na^+^ into the vacuole. It has been proposed that the SOS1 protein coordinates with high-affinity K^+^ transporters1 (HKT1) to adjust the distribution of Na^+^ between roots and shoots [14,15].

Plant responses to salt stress involve various mechanisms and processes that crosstalk with biotic stress responses [16]. This is the case for defense proteins, specifically class 10 of the pathogenesis-related (PR) proteins (PR10), which contribute to both abiotic and biotic stress responses [17,18]. The PR10 protein was first identified in parsley [19] and was subsequently described in various dicot and monocot plant species, including wheat [20], common bean [21], and soybean [22]. The classification of the PR10 family has been conducted in various plant species. Based on previous reports, the two major subgroups consist of the major latex proteins (MLP) as well as both the classical PR10 and the allergens Bet v1-like proteins [21,23,24]. The classical PR10 proteins are ribonucleases due to the conserved glycine-rich motif (GXGGXGXXK), which is also called the P-loop motif [25]. In the common bean, there are ten PvPR10 proteins that share this conserved motif, while PvMLP proteins lack this conserved motif, which is restricted to GXXXXXG [21]. Other functions attributed to PR10 proteins include antimicrobial activity [18,25,26], regulation of plant hormones via binding to various hormones like cytokinin and abscisic acid [27,28,29,30], control of flavonoid synthesis [31], and norcoclaurine synthase activity [32]. It has been reported that under biotic stress, the mode of action of PR10 proteins is associated with the ethylene (ET), jasmonate (JA), and salicylic acid (SA) pathways, as well as with transcription factors and effector proteins like leucine-rich repeat protein 1 (LRR1), leading to plant disease resistance [28,33]. During the infection of common beans with the hemibiotrophic fungus *Colletotrichum lindemuthianum*, PR10 proteins play a prominent role in coping with disease and interplay with ET, SA, and JA pathways [34].

Besides controlling plant growth and development, phytohormones mediate plant responses to salinity stress [35]. For instance, ABA and JA generally act as positive regulators of salt tolerance, whereas GA has negative regulatory effects [36]. The outcome of salt stress responses depends on a complex hormonal crosstalk that varies with plant growth stage and environmental context [35]. Various reports indicate that phytohormones are involved in signaling networks that include PR10 proteins, which contribute to plant responses against stresses caused by viruses, bacteria, fungi, and insects [20,28,37].

In our previous study, *PvPR10* gene expression was up-regulated by exogenous application of phytohormones, like SA, JA, and abscisic acid (ABA). Moreover, the majority of these genes were induced under salt stress, especially in roots [21]. However, little is known about the mode of action of common bean PvPR10 proteins under salt, phytohormones SA and JA, and the combined salt–SA/JA stresses. Thus, in this study, we aimed to understand the mechanism involved in PvPR10-mediated combined stress response through the physiological, biochemical, and molecular analyses of transgenic *Arabidopsis* plants expressing *PvPR10*.

## 2. Results

### 2.1. Response of Common Bean to Salt, Phytohormones, and Combined Salt–Phytohormone Stresses

**Figure 1:**
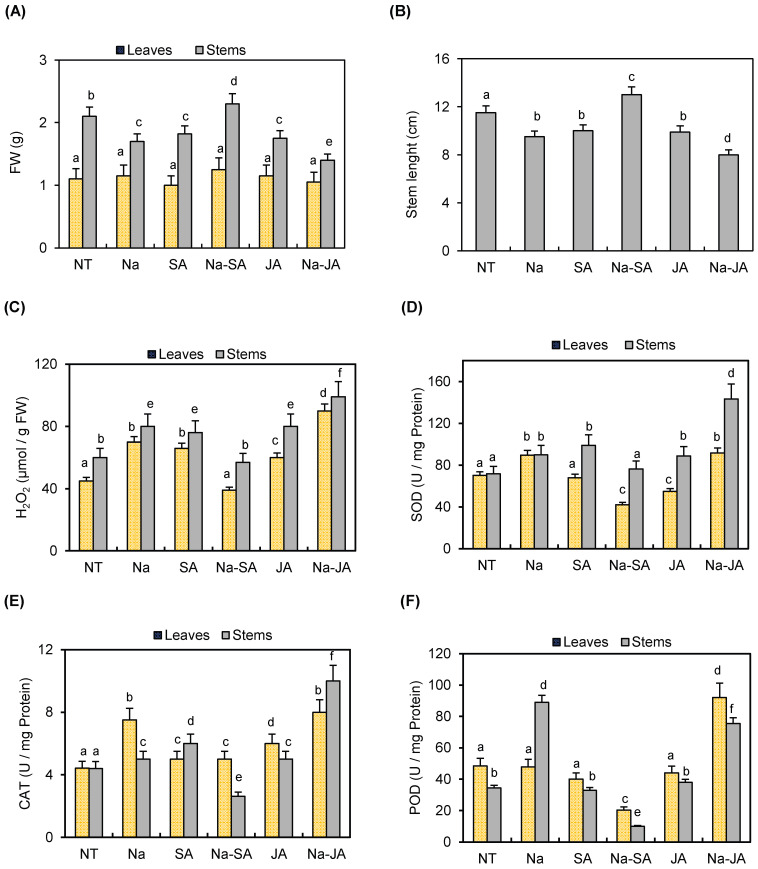
Effects of salt (Na), phytohormones SA and JA, and the combined treatments of salt with salicylic acid (Na–SA) and salt with jasmonic acid (Na–JA) on the aerial parts (leaves and stems) of common bean after 3 days of stress exposure. The analysis consisted of the measurement of (**A**) fresh weight (FW), (**B**) stem length, (**C**) H_2_O_2_ levels, and the activities of the three ROS-scavenging enzymes (**D**) SOD, (**E**) CAT, and (**F**) POD. NT: non-treated plants. Error bars represent the mean ± standard deviation of three biological replicates. Different letters indicate statistically significant differences between treatments (*p* < 0.05) based on post hoc comparisons.

The white-seeded common bean (*Phaseolus vulgaris*) cv. coco was exposed for 3 days to various stress conditions, including salt (NaCl) alone, the phytohormones SA or JA alone, and salt in combination with SA (NaCl–SA) or JA (NaCl–JA). Plant responses were evaluated by measuring fresh weight (FW), stem length, H_2_O_2_ levels, and the activity of the ROS-scavenging enzymes SOD, CAT, and POD in the aerial parts. Our results showed that none of these stresses affected leaf growth, whereas stem growth was reduced under salt stress, especially in combination with JA (NaCl–JA). Indeed, stem FW was reduced by salt and phytohormone treatments. Interestingly, this reduction was more pronounced under NaCl–JA compared to non-treated controls (Figure 1A). Moreover, stem length was significantly reduced by salt, phytohormones, and NaCl–JA. By contrast, the presence of SA improved the growth of stem tissues under salt stress (Figure 1B). Remarkably, high levels of H_2_O_2_ were detected in leaves and stems exposed to the combined NaCl–JA treatment compared to those obtained under single stress. In contrast, H_2_O_2_ levels remained stable in leaves and stems under the combined stress NaCl–SA compared to non-treated plants (Figure 1C). To better understand the response of the common bean to these stress conditions, we analyzed the activities of SOD, CAT, and POD, three key antioxidant enzymes. The analysis of SOD activity, the first line of enzymatic defense, showed an increase in leaves and stems under salt stress and NaCl–JA, while levels remained stable under NaCl–SA, especially in stems (Figure 1D). Under salt stress, CAT and POD activities increased in leaves and stems, respectively, compared to the non-treated plants. The application of JA with salt stress enhanced CAT activity in stems and POD activity in leaves, compared to the salt stress. However, the activity of these two H_2_O_2_-scavenging enzymes remained stable or decreased under the combined stress NaCl–SA. Likewise, the presence of SA or JA alone did not induce CAT and POD activities (Figure 1E,F). All these findings suggest that SA may mitigate salt stress in the aerial parts of the common bean, while JA accentuates the effect of salt stress, likely through the accumulation of H_2_O_2_ in aerial parts and induction of ROS-scavenging enzymes.

### 2.2. Expression Profile Analysis of PvPR10 Genes Under Salt, Phytohormones, and Combined Salt–Phytohormone Stresses

**Figure 2:**
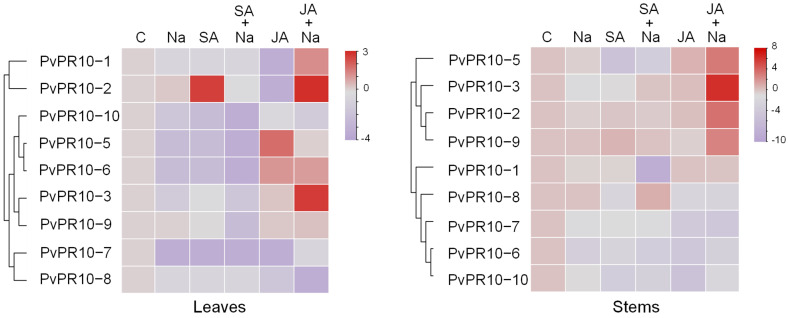
Expression profile of *PvPR10* genes in leaves and stems of common bean subjected for 3 days to various stress treatments, alone or in combination, as determined by qRT-PCR. Single stress treatments included salt (Na) and phytohormones (SA or JA). The combined stresses were the NaCl with SA (SA + Na) and NaCl with JA (JA + Na). Untreated plants served as controls (C). Expression data were converted to log_2_ for visualization. The heat map was drawn using TBtools-II software. The colors red and purple correspond to up-regulation and down-regulation of the corresponding genes, respectively. Depending on their expression profile, *PvPR10* genes were grouped into four subgroups in leaves, while they were grouped into two major subgroups in stems.

In the common bean, ten *PvPR10* genes were previously identified and classified as the second major subgroup of the PR10 family [21]. qRT-PCR analysis was carried out to analyze the expression profiles of *PvPR10* genes in leaves and stems of the common bean exposed to single stresses (salt, SA, or JA) or to combined stresses (NaCl–JA or NaCl–SA). As shown in Figure 2, in leaves, the application of exogenous SA induced the expression of *PvPR10-2*, while JA treatment enhanced the expression of *PvPR10-5* and *PvPR10-6*. Moreover, the combined NaCl–JA stress resulted in a significant induction of four genes in leaves—*PvPR10-1*, *PvPR10-2*, *PvPR10-3*, and *PvPR10-6*—whereas no up-regulation was observed under the combined NaCl–SA treatment (Figure 2). In stem tissues, the two genes *PvPR10-3* and *PvPR10-2* were also induced under NaCl–JA stress, along with *PvPR10-5* and *PvPR10-9*. By contrast, no significant induction of *PvPR10* genes was observed under salt, SA, JA, or NaCl–SA treatments in stem tissues. Taken together, these results show that the two genes *PvPR10-2* and *PvPR10-3* were induced under the combined stress NaCl–JA in both leaf and stem tissues, suggesting their potential role in response to this stress. According to our previous study, *PvPR10-3* is classified as a classic PR10 protein, while PvPR10-2 is a potential allergen [21]. In general, the classic PR10 proteins are crucial in response to abiotic and biotic stresses [25,38]; thus, we focused in this study on the analysis of *PvPR10-3*’s role in response to various stress treatments.

### 2.3. Generation of PvPR10-3-Expressing Arabidopsis Lines

To gain insight into the role of *PvPR10-3* in response to salt and phytohormone treatment, its corresponding cDNA was cloned into the binary vector pBI321 under the control of the duplicated cauliflower mosaic virus 35S promoter (P35S) (Supplementary Figure S1A). Ten *Arabidopsis* plants were transformed with the *A. tumefaciens* GV3101 strain harboring the pBI321-*PvPR10-3* construct. Transformed seedlings were selected on MS medium containing 20 mg L^−1^ hygromycin, resulting in six transformed plants. The presence and expression of *PvPR10-3* were verified using PCR and RT-PCR, respectively (Supplementary Figure S1B,C). As expected, *PvPR10-3* was not detected in wild-type (Wt) plants but was expressed in all these transgenic lines, with expression particularly high in lines A1, A2, and A3. In addition, in these three lines, the hygromycin resistance marker (*hptII*) segregated as a single copy gene based on a 3:1 resistance: sensitivity ratio. Thus, the two lines A1 and A3 were used to perform physiological and molecular analyses.

### 2.4. Enhanced JA-Mediated Salt Stress Tolerance in PvPR10-3 Arabidopsis Lines Is Linked to Reduced H_2_O_2_ Accumulation

**Figure 3:**
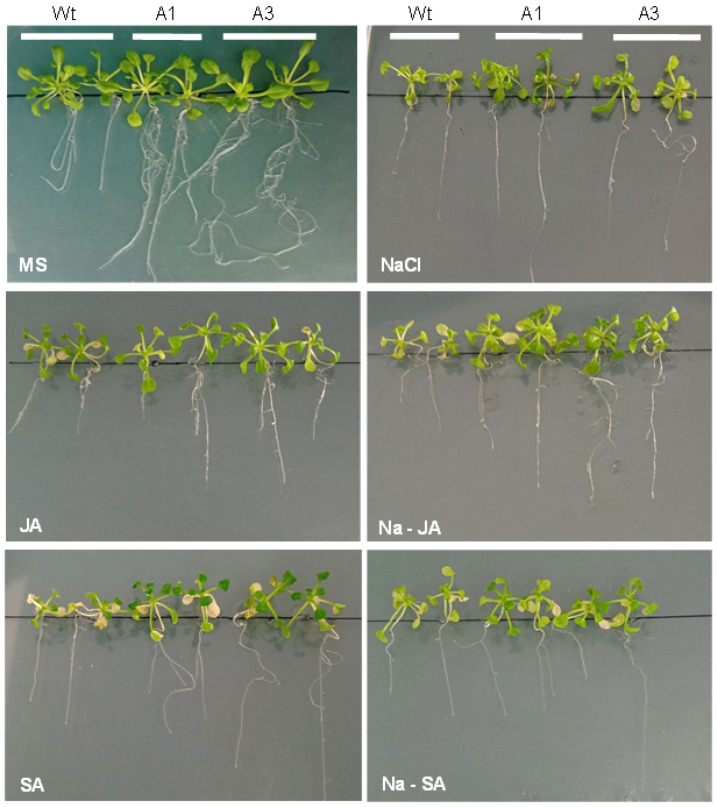
Phenotypic response of the transgenic *PvPR10-3*-expressing *Arabidopsis* lines A1 and A3, and Wt plants to salt, phytohormones SA and JA, and combined stresses. Seven-day-old seedlings were placed on MS medium containing 100 mM NaCl, JA, SA, NaCl with JA (Na–JA), and NaCl with SA (Na–SA) or maintained on MS medium alone (MS), and grown for an additional 10 days.

The response of *PvPR10-3*-expressing *Arabidopsis* lines to salt stress (100 mM NaCl), phytohormones SA or JA alone, and combined stresses (NaCl–SA and NaCl–JA) was evaluated and compared to non-transformed wild-type (Wt) plants. As shown in Figure 3, under non-stress conditions, transgenic plants and Wt plants exhibited a similar aerial growth phenotype; however, the roots of transgenic plants appeared much more developed. The presence of salt in the medium limited the growth of all the seedlings; however, growth inhibition was more pronounced in Wt than in transgenic lines, suggesting improved salt tolerance in the latter. Moreover, the two transgenic lines exhibited tolerance to the application of SA and JA compared to the Wt plants. This trend was further enhanced in the presence of JA, where NaCl–JA treatment promoted the growth of all lines, with A1 and A3 still displaying superior growth compared to Wt. Conversely, the combination of NaCl with SA was less effective in mitigating salt-induced growth inhibition in both Wt and transgenic lines (Figure 3).

**Figure 4:**
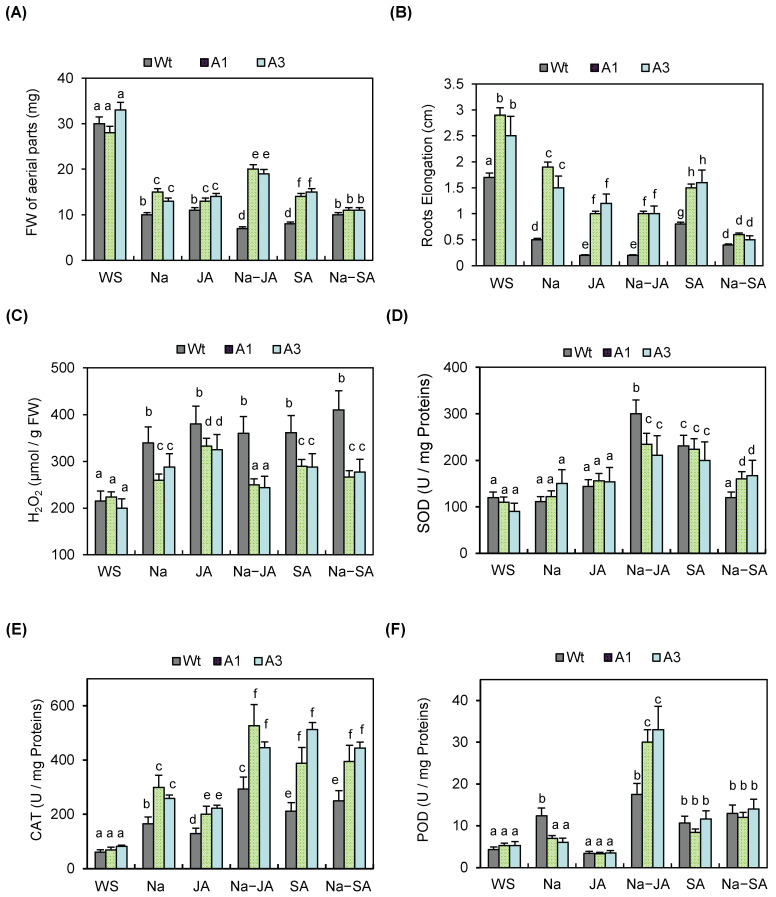
Response of Wt and transgenic *PvPR10-3*-expressing lines A1 and A3 to salt, phytohormones, and combined stresses. The following parameters were measured: (**A**) fresh weight (FW) of aerial parts; (**B**) root elongation; (**C**) H_2_O_2_ levels as indicator of redox homeostasis; (**D**) SOD; (**E**) CAT; and (**F**) POD activity in leaves of WT and transgenic lines. Plants were grown under the following conditions: normal growth condition (WS: no stress), salt stress (Na), the phytohormones JA or SA, and combined stresses NaCl–JA (Na–JA) and NaCl–SA (Na–SA). Data represent the mean ± standard deviation of three biological replicates. Different letters above bars indicate statistically significant differences (*p* < 0.05) among treatments within each panel.

These findings were confirmed by determining the fresh weight (FW) of the aerial tissues of all these seedlings under normal and stress conditions. Salt stress caused a reduction in FW in all genotypes, with a more severe decline (~75%) in Wt plants than in transgenic lines (~50%). While SA co-treatment did not significantly improve FW in transgenic lines, JA application under salt stress partially alleviated growth inhibition, leading to increased FW in A1 and A3 relative to salt stress alone (Figure 4A).

Moreover, root elongation of these plants was analyzed under normal and stress conditions. Under normal conditions, root elongation of the transgenic lines was more pronounced than that of Wt plants. The application of stress treatments restricted the root elongation not only of the transgenic lines but also of the Wt plants. Interestingly, the transgenic lines displayed a higher growth rate under each stress condition compared to the Wt plants (Figure 4B).

To further investigate the basis of this enhanced tolerance, H_2_O_2_ accumulation in leaf tissues was assessed (Figure 4C). Indeed, the two lines A1 and A3 accumulated lower amounts of H_2_O_2_ compared to the Wt plants under salt and phytohormone stresses. Interestingly, under the combined stress NaCl–JA, the amounts of H_2_O_2_ of these lines were further reduced compared to both Wt plants and the same lines exposed to salt stress and JA treatment. In contrast, the A1 and A3 transgenic lines did not show such an increase: their H_2_O_2_ levels under NaCl + SA remained similar to those observed under NaCl or SA alone, and significantly lower than Wt. The activity of three antioxidant enzymes—SOD, CAT, and POD—was analyzed in these lines under these same stress conditions. The results showed that under salt stress and in the presence of JA or SA alone, only CAT activity was induced in the two transgenic lines compared to the non-treated plants, and it was higher than in Wt plants (Figure 4E). Notably, under combined NaCl–JA treatment, both CAT and POD activities increased significantly in the transgenic lines, exceeding levels in Wt plants. CAT activity was enhanced approximately twofold, and POD activity threefold, compared to the same lines under salt stress alone. Compared to JA alone, CAT and POD activities increased in the transgenic lines under NaCl–JA stress about 2.5-fold and 7-fold, respectively (Figure 4E,F). Under NaCl–SA stress, SOD and CAT activities were induced in the transgenic lines relative to Wt; however, there was no induction compared to stress with SA alone (Figure 4D,E).

Taken together, these results suggest that *PvPR10-3* expression improves salt and phytohormone tolerance in *Arabidopsis*, particularly when co-treated with JA, likely by upregulating CAT and POD activity to limit H_2_O_2_ accumulation and maintain redox homeostasis.

### 2.5. Enhanced Salt and JA Stress Resilience in PvPR10-3-Expressing Lines Involves ABA-Dependent and Independent Pathways and Phenolic Accumulation

To investigate the physiological and molecular mechanisms underlying the response of *PvPR10-3*-expressing *Arabidopsis* lines to salt and combined salt-phytohormone stresses, 30-day-old seedlings of the transgenic lines A1 and A3, along with Wt plants, were irrigated with salt solution (150 mM NaCl) and either sprayed with SA or JA or left untreated for 10 days.

**Figure 5:**
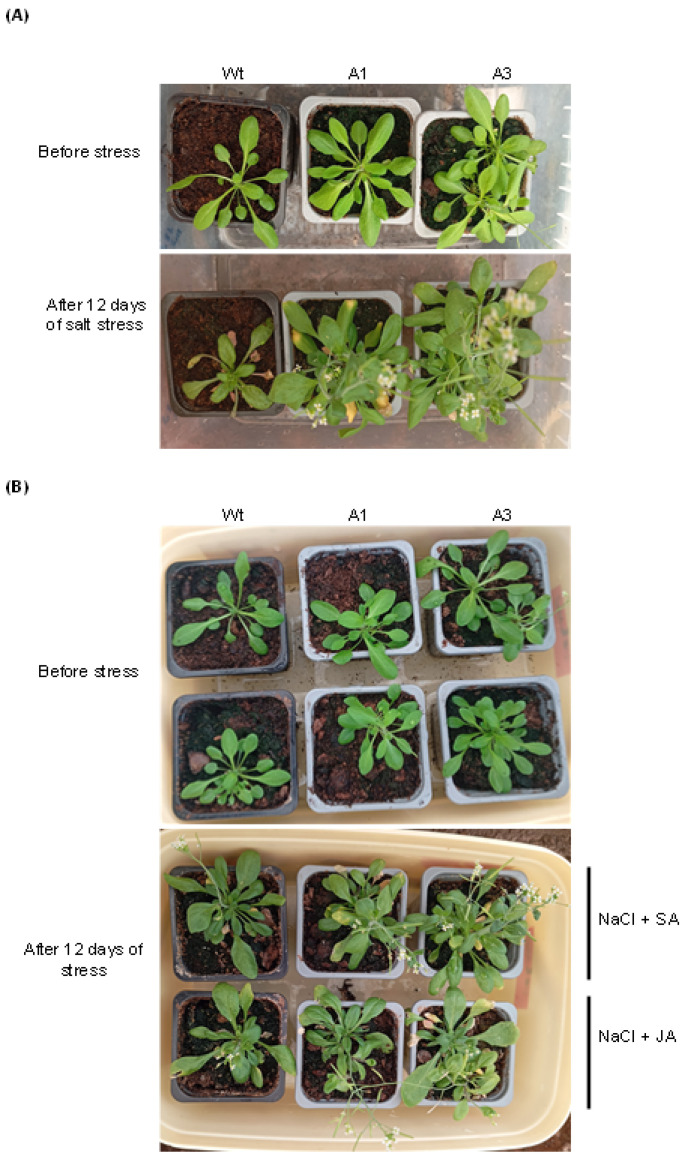
Response of *PvPR10-3*-expressing *Arabidopsis* lines to either salt stress (**A**) (150 mM NaCl) or combined stress (**B**) (NaCl + JA) or SA (NaCl + SA). 30-day-old seedlings of transgenic lines (A1 and A3) and Wt plants were irrigated for 10 days with salt solution (150 mM NaCl) and sprayed either with distilled water (only salt stress) (**A**) or with phytohormone solutions (100 µM SA or 100 µM JA) (**B**). The seedlings used for salt stress (**A**) and the two combined stresses (**B**) were photographed before (upper panels) and after (lower panels) the application of stress treatments. All treatments were performed at the same time.

As shown in Figure 5A, the transgenic lines showed more vigorous growth than the Wt plants with progressively more developed aerial parts over time under salt stress. Moreover, when exposed to JA with salt stress, the transgenic lines exhibited better growth than the Wt plants, which was not the case under the combined stress NaCl–SA (Figure 5B), suggesting that JA contributed to salt stress resilience of the transgenic lines.

**Figure 6:**
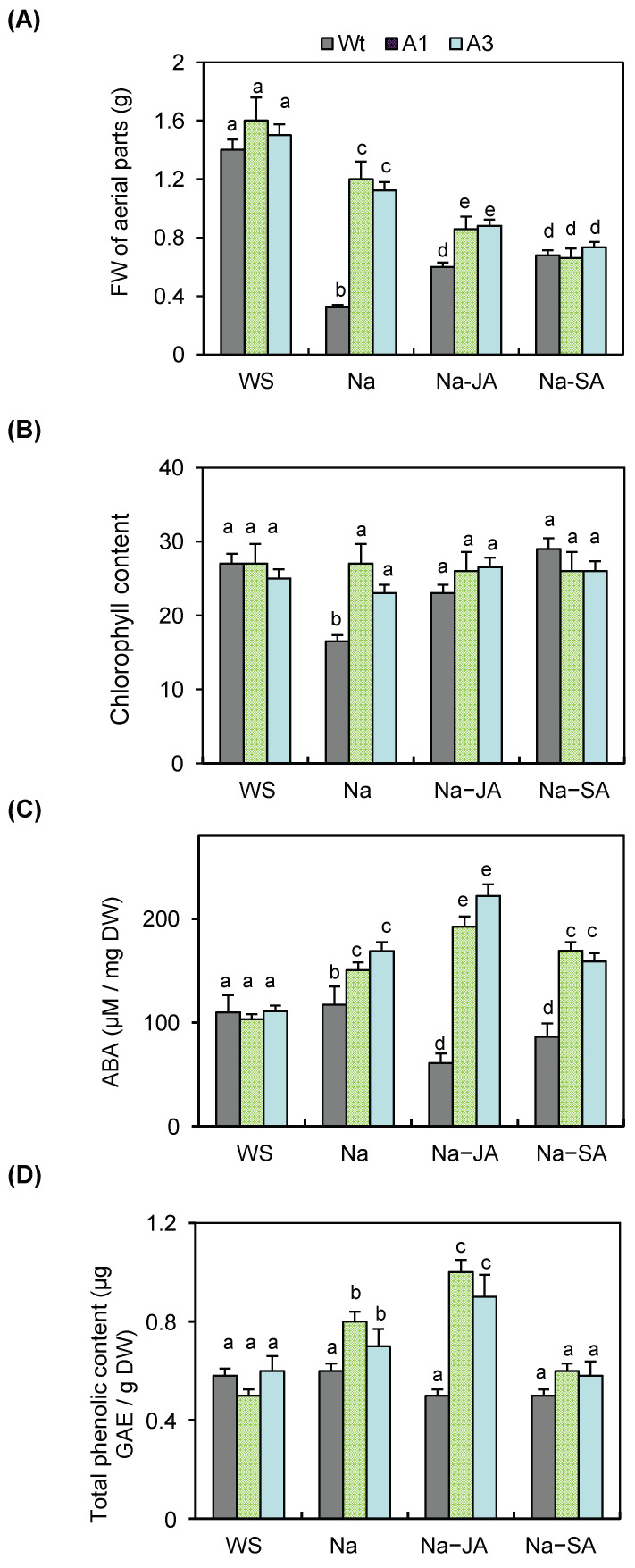
Analysis of physiological parameters in wild-type (Wt) and *PvPR10-3*-expressing *Arabidopsis* lines. Determination of (**A**) fresh weight (FW), (**B**) chlorophyll content, (**C**) ABA content, and (**D**) phenolic compound levels in the aerial parts of the transgenic lines A1 and A3 and the non-transformed Wt plants exposed to salt stress (150 mM), associated or not with phytohormones JA (Na–JA) or SA (Na–SA). WS: without stress. Data represent the mean ± standard deviation of three biological replicates. Different letters above bars indicate statistically significant differences (*p* < 0.05) among treatments within each panel.

To strengthen these observations, fresh weight (FW), chlorophyll content, reproductive output, ABA, and phenolic compound levels were measured under each treatment (Figure 6A–D). Under salt stress alone, the FW of the two lines was higher than that of Wt plants (Figure 6A); however, when NaCl was combined with either JA or SA, all lines exhibited reduced FW, and differences between transgenics and Wt became less pronounced under NaCl–SA stress, suggesting that the role of *PvPR10-3* was attenuated under this combined stress. By contrast, the FW of the two lines was higher than that of the Wt plants under NaCl–JA (Figure 6A). Chlorophyll content followed a similar trend (Figure 6B): it was significantly reduced in Wt under salt stress but remained stable in the transgenic lines, reflecting better photosynthetic maintenance. Under combined treatments (NaCl–JA or NaCl–SA), chlorophyll content was preserved at similar levels across all genotypes, indicating a general stabilizing effect of JA and SA on pigment maintenance.

ABA accumulation (Figure 6C) was also enhanced in A1 and A3 under salt stress compared to Wt. Notably, exogenous JA accentuated ABA production in the two transgenic lines, while under the combined stress, NaCl–SA ABA content was almost similar to that under salt stress (Figure 6C).

The transgenic lines A1 and A3 showed a higher accumulation of phenolic compounds under salt stress compared to wild-type (Wt) plants. Foliar application of JA further enhanced this accumulation, whereas SA had no significant effect (Figure 6D), suggesting that JA may contribute to the activation of the phenylpropanoid metabolism leading to stress resilience of the transgenic lines. This hypothesis is consistent with previous studies linking JA signaling and secondary metabolite production to abiotic stress adaptation [39].

**Figure 7:**
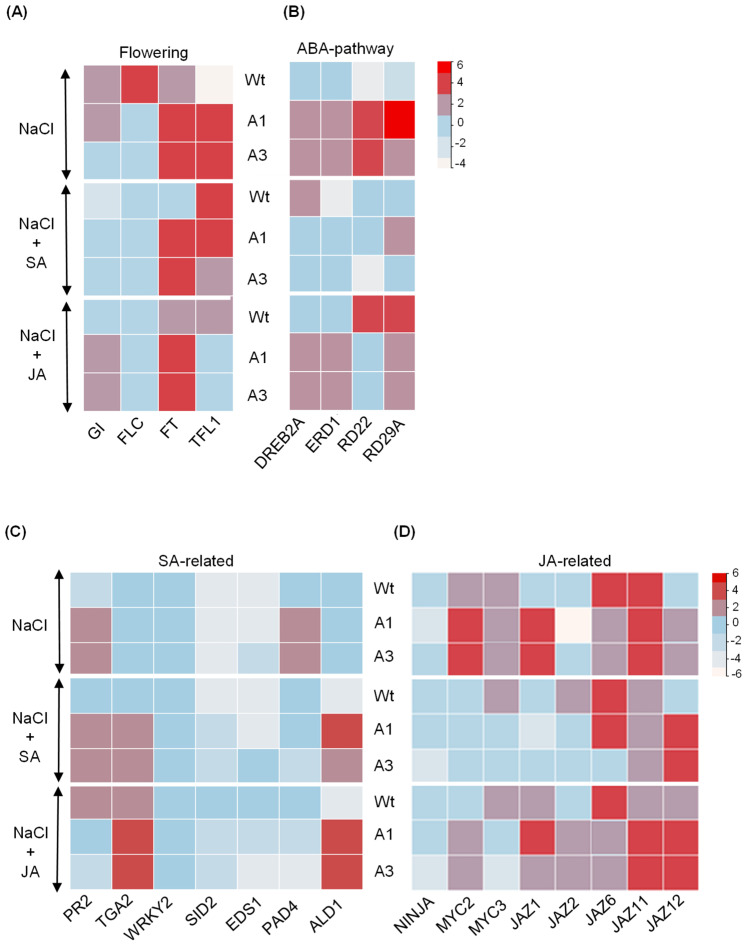
Expression profile analysis of a subset of genes related to (**A**) flowering, (**B**) ABA, (**C**) SA, and (**D**) JA signaling pathways. qRT-PCR analysis was performed on Wt plants, and *PvPR10-3*-expressing transgenic lines A1 and A3 under salt stress (NaCl), and the two combined stresses NaCl–SA and NaCl–JA. Data from qRT-PCR reactions were converted to log_2_ to be presented. The log_2_ value of the relative expression of each gene under control condition (without stress) was zero. Heat map was drawn using TBtools-II software. The colors red and white correspond to the up-regulation and down-regulation of the corresponding gene, respectively. The primers used for qRT-PCR analyses are detailed in Supplementary Table S1.

To further elucidate the mechanisms underlying *PvPR10-3*-mediated stress responses, we analyzed the expression profiles of flowering-related genes, including *GIGANTEA* (*GI*), *FLOWERING LOCUS T* (*FT*), *FLOWERING LOCUS C* (*FLC*), and *TERMINAL FLOWER LOCUS 1* (*TFL1*), in Wt and the transgenic lines exposed to salt and to the two combined stresses. Under salt stress and combined NaCl–SA stress, *FT* and *TFL* were up-regulated in the transgenic lines. By contrast, the combined stress NaCl–JA enhanced the expression of *GI* and *FT* genes (Figure 7A).

We also analyzed the expression of genes involved in ABA signaling, including DEHYDRATION RESPONSIVE ELEMENT BINDING (DREB2A), EARLY RESPONSIVE TO DEHYDRATION (ERD1), RESPONSE TO DESICCATION (RD29A), and RESPONSIVE TO DEHYDRATION 22 (RD22), in seedlings exposed to the same stresses. qRT-PCR analysis showed that under salt stress, all four genes were significantly up-regulated in transgenic lines compared to Wt, but surprisingly down-regulated under combined NaCl–SA stress. Notably, in the NaCl–JA treatment, DREB2A and ERD1 remained highly induced in the transgenic lines, while RD22 and RD29A showed only weak or moderate expression. Thus, these results suggest that *PvPR10-3* contributes to salt and JA responses by preferentially activating genes associated with the ABA-independent signaling pathway.

These results demonstrate that *PvPR10-3* confers resilience to salt and JA stress by activating an ABA-independent signaling pathway, promoting the accumulation of protective phenolic compounds and activation of some flowering-related genes, including *FT* and *GI* genes.

### 2.6. Crosstalk Between JA and SA During Salt Stress Response of PvPR10-3-Expressing Arabidopsis Lines

To investigate the molecular mechanisms underlying salt stress responses in *PvPR10-3* overexpressing *Arabidopsis* lines, we analyzed the expression of several genes involved in SA- and JA-related defense pathways. For the SA pathway, we examined genes involved in SA biosynthesis (e.g., *SID2*), SA accumulation and signaling (*EDS1, PAD4, ALD1*), and downstream defense response (*PR2,* transcription factors *TGA2* and *WRKY2*) (Figure 7C). Under salt stress, only *PAD4* and *PR2* were significantly upregulated in both transgenic lines. Under NaCl–SA and NaCl–JA, *TGA2* and *ALD1* were consistently upregulated in A1 and A3 compared to Wt, suggesting that these genes may contribute to crosstalk between SA and JA signaling under salt stress. Additionally, we analyzed genes in the JA pathway, including the co-repressor *NINJA,* the transcription factors *MYC2* and *MYC3*, and members of the JAZ family (JAZ1, JAZ2, JAZ6, JAZ11, and JAZ12) [40]. We found that salt stress strongly induced *MYC2*, *MYC3*, and *JAZ1* in both transgenic lines, while *JAZ12* was selectively upregulated under NaCl–SA. Under NaCl–JA, *JAZ2* and *JAZ12* showed enhanced expression, while *MYC3* was repressed compared to salt stress alone. NINJA expression was modestly increased in A1 and A3 under NaCl–JA but not consistently across all treatments (Figure 7D). Taken together, these results suggest that the response to salt stress with JA enhanced the expression of multiple genes related not only to JA but also to SA, including *TGA2* and *ALD1*.

## 3. Discussion

The common bean (*Phaseolus vulgaris* L.) is one of the most important food legumes worldwide because of its high amount of proteins, as well as carbohydrates, vitamins, minerals, dietary fibers, and phenolic compounds [41]. Abiotic stress, like drought, affects more than 60% of dry bean production worldwide [42]. The abiotic stress resilience of the common bean results from complex interactions among physiological, biochemical, and genetic processes [42]. In general, phytohormones are essential not only for growth and development but also for their antagonistic or synergistic actions to help plants mitigate stress [43,44].

This study provides a comprehensive physiological and molecular characterization of the role of a common bean PR10 protein, *PvPR10-3*, in mediating salt stress tolerance, with a particular emphasis on its synergistic interaction with JA and its contrasting relationship with SA. Our findings position *PvPR10-3* as a central node in a hormone signaling network that coordinates antioxidant defense, secondary metabolism, and ABA-independent stress signaling to enhance resilience.

To the best of our knowledge, little is known about the role of PvPR10 proteins in the response of common beans to the combined salt–phytohormone (SA or JA) stress. Previously, we have demonstrated the importance of these proteins in the response of common beans to several abiotic stress and phytohormone treatments [21]. In the present study, we found that the expression of several *PvPR10* genes, especially *PvPR10-2* and *PvPR10-3* was up-regulated in leaves and stems of common beans subjected to salt stress in combination with exogenous jasmonate (NaCl–JA), suggesting that these two genes are prominent candidate genes in response to salt and JA treatments. Our results show that the association between salt and JA application reduces stem growth and increases the oxidative status of the aerial parts of common beans compared to salt stress. Nevertheless, the exogenous SA application reduced the effect of salt stress by enhancing stem tissue growth and reducing H_2_O_2_ accumulation, while *PvPR10* genes were down-regulated under this combined stress. In concordance with our findings, the beneficial effect of SA has been observed in the annual herbaceous legume, cowpea plants, and rice via the activation of morphological, biochemical, and physiological processes [45,46]. Furthermore, the ROS-scavenging enzymes in leaves and stems of common beans were not activated under the combined stress NaCl–SA compared to the NaCl treatment. This result suggested that the supplementation of SA could restrict the uptake of the ion Na^+^ in the plant cells, similarly to that in rice [46].

The accumulation of H_2_O_2_ under NaCl–JA stress in the common bean indicates a significant oxidative challenge, which is a primary cause of the anatomical and physiological disruptions observed in salt-stressed plants. Salt stress-induced oxidative damage is a well-documented phenomenon, where ion imbalance leads to metabolic disorders and overproduction of ROS, causing peroxidation of lipids, protein degradation, and nucleic acid damage [47]. Our observation that the transgenic *Arabidopsis* lines expressing *PvPR10-3* exhibited lower H_2_O_2_ accumulation under stress underscores the protein’s role in reducing oxidative damage. The enhanced activity of key antioxidant enzymes like CAT and POD in these lines, particularly under NaCl–JA treatment, points to a mechanism where *PvPR10-3* potentiates the JA-induced activation of the enzymatic ROS-scavenging machinery to maintain cellular redox homeostasis. This robust biochemical response is crucial for maintaining cellular integrity and preventing the anatomical and physiological damage that would otherwise limit plant growth.

In addition to SA, various reports demonstrate that the exogenous JA plays a major role in inhibiting the negative effect of salt stress [48,49,50]. However, our findings showed that the application of JA exacerbated the negative effect of salt stress compared to the exogenous SA, suggesting that the variation of JA levels in common beans could restrict the mechanism of salt stress tolerance.

So far, little information is available regarding the role of the *PvPR10-3* gene in the response of the common bean to salt stress, associated or not with the exogenous JA or SA. Thus, it was functionally characterized in *Arabidopsis* plants to better understand its role in response to salt associated with the foliar application of JA. Our results showed that the expression of *PvPR10-3* in *Arabidopsis* exhibited salt and phytohormone tolerance in the transgenic lines. Similar to our findings, the expression of the members of the PR10 family of common bean, PvMLP19 and PR10a of potato, contributes to salt tolerance of the transgenic *Arabidopsis thaliana* and *Solanum tuberosum*, respectively [51,52]. Interestingly, tolerance of the transgenic lines to salt and SA or JA treatments was associated with a better maintenance of chlorophyll content. The preservation of chloroplastic pigments is a key indicator of photosynthetic integrity, as salt stress severely impacts the photosynthetic machinery through both stomatal limitations, which reduce CO_2_ availability, and non-stomatal effects, including degradation of photosynthetic pigments and inhibition of electron transport from PSI and PSII [53]. Our finding that chlorophyll content was significantly preserved in the *PvPR10-3* transgenic lines under salt stress suggests a robust protection of the photosynthetic apparatus against these damaging effects. Similarly, the plant growth regulator 24-Epibrassinolide limits the chlorophyll degradation in tomato plants, resulting in salt stress tolerance [54]. The stability of chlorophyll levels in our transgenic plants, coupled with the observed reduction in H_2_O_2_, indicates that *PvPR10-3* helps maintain photosynthetic function by mitigating the oxidative stress that leads to pigment loss and photosystem damage. Furthermore, we demonstrated that JA application improved salt stress tolerance of the transgenic lines by reducing oxidative damage, as manifested by low levels of H_2_O_2_, and promoting the activity of ROS-scavenging enzymes, especially CAT and POD. Our results were in line with the study of Qui et al. [55], who also demonstrated that foliar application of JA protects wheat from salt stress through the activation of antioxidant enzymes and nonenzymatic antioxidants. Moreover, JA interacts with other phytohormones to regulate ROS homeostasis via activation of the ROS-scavenging system to lead to salt tolerance [50,56]. For instance, MeJA cooperates with SA and protects soybean from the harmful effects of salt stress [57]. In general, SA and JA-mediated defense response pathways are mutually antagonistic during response to biotrophic and necrotrophic pathogens, respectively [58].

**Figure 8:**
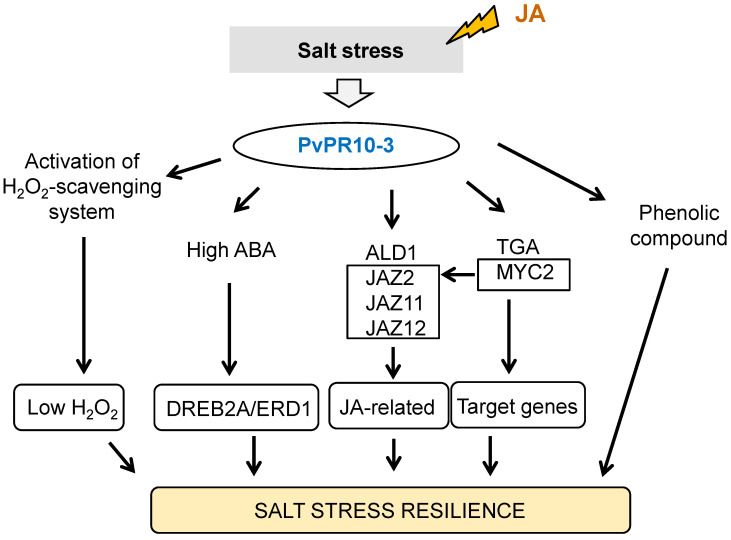
Proposed model for *PvPR10-3-*mediated tolerance to combined salt and JA stress in *Arabidopsis*. Our results suggest that the phytohormone JA enhances salt stress resilience of *PvPR10-3*-expressing *Arabidopsis* plants via the activation of H_2_O_2_-scavenging enzymes CAT and POD, high accumulation of ABA, and phenolic compounds. Despite the accumulation of ABA, the response to this combined stress is ABA-independent via the activation of DREB2A and ERD1 components. Moreover, several components of the JA signaling pathway are induced in response to this combined stress, such as the transcription factor MYC2 and the three JAZ proteins JAZ2, JAZ11, and JAZ12. Interestingly, JA crosstalks with the SA signaling pathway via the potential involvement of the transcription factor TGA2 and the ALD1 that controls the SA accumulation.

To boost our understanding of the mechanism underlying the role of *PvPR10-3* protein in salt–JA crosstalk, physiological and molecular analyses were conducted and then compared to those under NaCl and NaCl–SA treatments. Interestingly, the data showed that contrary to SA, the exogenous JA enhanced the salt tolerance phenotype of the *PvPR10-3*-expressing *Arabidopsis* lines due to the enhancement of ABA and phenolic compounds production. In the same way, many findings show that JA application enhances ABA accumulation to promote salt stress response [59,60]. ABA plays a major role in response to salt stress by absorbing stress signals and regulating various physiological and biochemical processes, including the modulation of stomatal aperture, root architecture, and the antioxidant defense systems [61,62]. Additionally, ABA interacts with various other phytohormones like auxin, cytokinin, SA, and JA to enhance the plant’s ability to undergo abiotic stress [50,61]. Several studies suggest that ABA and JA contribute to salt stress tolerance in a cooperative way for regulating antioxidant status and the expression of many target genes [63]. Our results suggested that *PvPR10-3* proteins confer salt stress tolerance of *Arabidopsis* plants via the possible coordination between JA and ABA. Furthermore, we showed that the expression of the two ABA-independent genes *AtDREB2A* and *AtERD1* was induced in transgenic lines under the combined NaCl–JA stress, while the presence of SA down-regulated their expression, suggesting that JA contributes to salt tolerance of the transgenic lines via the induction of the ABA-independent pathway. *AtERD1* is regarded as a typical stress response gene marker [64]. In addition to this gene, *AtDREB2A* is crucial for drought stress tolerance of transgenic sugarcane [65]. On the other hand, we speculated that the exogenous JA enhances salt stress tolerance of the transgenic lines via the induction of *GI* and *FT* genes under NaCl–JA treatment compared to NaCl stress. GI is involved not only in regulating the flowering time and circadian clock resetting but also in abiotic stress tolerance [66]. The accumulation of ABA promotes flowering via upregulation of *FT* to reduce the drought effect [67]. Similar to our findings, JA caused the ABA accumulation in the transgenic lines and conferred salt tolerance via the induction of the *FT* gene, which promotes flowering in plants [68]. Thus, this highlights the possible molecular crosstalk between the circadian clock, flowering, ABA, and JA signaling pathways to cope with salt stress via the involvement of *PvPR10-3* protein (Figure 8).

Moreover, the data showed that JA coordinates with two components of the SA pathway, which are the transcription factor TGA2 and the gene related to SA accumulation *ALD1*, to improve salt stress tolerance of the transgenic lines. TGA transcription factors play a pivotal role in responding to abiotic and biotic stresses through the regulation of target gene expression and participation in several biological regulatory processes [69]. Furthermore, the JA-related genes induced during the response of transgenic lines to NaCl–JA were *MYC2*, *JAZ2*, *JAZ11*, and *JAZ12* (Figure 8). MYC2 is a major regulator of the JA signaling pathway during abiotic and biotic stresses, plant growth and development, and specific metabolite synthesis [70]. Except *JAZ12* gene, these genes were not induced in the transgenic lines in response to NaCl–SA stress. Thus, we suggested that the transcription factors MYC2 and TGA2, SA-related component ALD1, and JA signaling components are the main nodes in the crosstalk of JA with the other phytohormones.

Interestingly, tolerance of the transgenic lines to salt stress was manifested by phenolic compounds, especially in the presence of JA, in order to better prevent stress and to regulate some physiological activity [39]. Moreover, many functions are attributed to the phenolic compounds under abiotic stress, including harmful ROS scavenging, membrane stabilization, and osmotic adjustment [71]. Various studies report that JA regulates the biosynthesis of phenolic compounds [72,73,74]. Here, we showed that the accumulation of phenolic compounds is associated with the exogenous application of JA to salt stress, leading to salt stress tolerance, which is not the case in the presence of SA. On the other hand, it has been reported that multiple transcription factors, like bHLH and MYB, modulate the expression of genes involved in bioactive compounds synthesis [75]. Here, we demonstrated that the bHLH transcription factor MYC2 is induced in response to salt stress associated with JA in the transgenic lines, suggesting its possible role in controlling the synthesis of the bioactive compounds during the combined NaCl–JA stress. It is worth mentioning that the response to salt stress associated with the exogenous phytohormone JA is complex and implicates various signaling pathways and components to cope with stress. So, other studies are needed to better understand the cross-coordination between JA and other phytohormones and their orchestration by the *PvPR10-3* protein during salt stress response.

## 4. Materials and Methods

### 4.1. Plant Material and Treatments

The plant material used in this study is the white-seeded common bean (*Phaseolus vulgaris*), cv. coco, which were surface-sterilized with 95% alcohol and then with 0.2% mercuric chloride. After germination, seedlings were grown for 2 weeks in a mixture of peat/perlite (2/1) in a greenhouse with temperatures of 24 °C during the day and 18 °C at night, and relative humidity in the range of 60 to 70% [21]. Subsequently, salt stress was applied by irrigation every two days with salt solution (150 mM NaCl), while phytohormones SA (0.25 mM) and JA (0.25 mM) were applied by spraying, as described by Feki et al. [21]. The concentrations of salt solution and phytohormones were used as mentioned previously by Feki et al. [21]. The two combined stresses were salt (150 mM NaCl) with 0.25 mM SA (NaCl–SA) or with 0.25 mM JA (NaCl–JA). All these stresses were applied for three days according to Feki et al. [21]. Control seedlings were irrigated and sprayed with distilled water. After three days of stress treatment, leaves and stem tissues of stressed and control plants were collected and immediately frozen in liquid nitrogen for total RNA extraction and also used for total protein extraction. For each stress treatment, we performed three biological replicates, and each replicate sample consisted of four pooled plants that were cultivated in the same conditions.

### 4.2. Isolation and Cloning of PvPR10-3 Gene

It has been shown previously that *PvPR10-3* is induced in roots and leaves of common beans exposed to salt stress (150 mM NaCl) for one day [21]. To amplify the ORF of *PvPR10-3*, we used as a template the cDNA from the leaves treated with salt stress for one day and using specific primers PR10-F and PR10-R (Table S1), which harbor the restriction sites X*ba*I and B*amH*I, respectively. The amplified fragment (471 pb) was cloned into pGMTeasy (Promega) and then entirely sequenced. After that, this recombinant vector was digested with X*ba*I and B*amH*I to release the ORF of *PvPR10-3* and to clone it into pBI321 binary vector into the same restriction sites. The resultant recombinant binary vector, called pBI321-PR10, contains the ORF of *PvPR10-3* cloned into the downstream of the constitutive cauliflower mosaic virus (CaMV) 35S promoter.

### 4.3. Arabidopsis Transformation and Selection of the Transgenic Lines

Firstly, the recombinant binary vector pBI321-*PvPR10-3* was introduced into the *Agrobacterium tumefaciens* strain GV3101 in order to transform *Arabidopsis thaliana* plants using the floral dipping technique. About six transformed plants were obtained by selection on Murashige and Skoog (MS) agar medium containing 20 mg L^−1^ hygromycin [76]. The presence of *PvPR10-3* in the genome of these six lines was verified through PCR amplification with the specific primers PR10-F and PR10-R. Moreover, the expression of the transgene *PvPR10-3* was analyzed via RT-PCR reactions using the *Arabidopsis actin* gene as an internal control (Table S1).

### 4.4. Response of the Transgenic Arabidopsis Plants to Salt and Combined Salt–Phytohormone Stresses

The seeds of the homozygous T3 transgenic *Arabidopsis* plants were sterilized and sown onto solid MS medium plates and allowed to grow in a 22 °C growth chamber under 16 h light and 8 h dark. After growing on MS medium for 7 days, the seedlings were transferred to MS medium containing or not 150 mM NaCl (salt stress), 150 mM NaCl with 100 µM JA (NaCl–JA), and 150 mM NaCl with 100 µM SA (NaCl–SA) and grown vertically for 10 days. The response of these transgenic lines was compared to that of the non-transformed (Wt) plants. In parallel, we performed stress treatments in pots by transferring 7-day-old seedlings into the soil and growing them for one month. After that, salt stress was applied by irrigation with a salt solution of 150 mM NaCl every two days. At the same time, the foliar parts of transgenic lines and Wt were sprayed with distillated water, to be used as control plants, or with two different phytohormones SA (100 µM) and JA (100 µM) every two days. The concentration of SA was chosen based on the study of Pasternak et al. [77], which demonstrates that the concentration of SA higher than 50 µM causes stress hormones. Concerning JA concentration, we used 100 µM, as described by Huang et al. [78]. The response to these stress treatments was evaluated for 10 days through the determination of the fresh weight of the aerial parts, the chlorophyll content, and ABA and phenolic compounds contents.

### 4.5. Analysis of Enzymatic ROS-Scavenging System

Total protein was extracted from aliquots of fresh tissues (250 mg) from both *Arabidopsis* seedlings, exposed or not to salt and combined stress, and common bean plants exposed to salt, phytohormones (SA and JA), and combined stress using the previously described protocol [79]. After the determination of protein concentration of each sample [80], the activities of the three major ROS-scavenging enzymes, CAT, POD, and SOD, were evaluated and then expressed as units mg^−1^ protein. One unit of CAT was defined as one μmol ml^−1^ H_2_O_2_ decomposed per minute. CAT activity was evaluated by monitoring the disappearance of H_2_O_2_ [81]. One enzyme unit of POD is defined as a change in one unit of absorbance min^−1^. POD activity was determined using the guaiacol oxidation method [82]. One unit of SOD was defined as the amount of enzyme that causes 50% inhibition of the reduction in NBT. SOD activity was determined based on the photochemical of nitro blue tetrazolium (NBT) method [83].

### 4.6. Determination of H_2_O_2_ and Chlorophyll Content

To determine the H_2_O_2_ amount in leaves of the transformed *Arabidopsis* lines and Wt plants exposed to salt and combined stress, and also in the aerial parts of common beans exposed or not to different stress conditions, fresh tissues (100 mg) were homogenized with 0.1% (*w*/*v*) trichloroacetic acid (TCA). Subsequently, the recuperated supernatant in each sample (500 µL) obtained by centrifugation at 12,000 rpm for 15 min was used to quantify the H_2_O_2_ levels [79].

The chlorophyll contents in leaves of transformed *Arabidopsis* lines and Wt plants exposed or not to salt stress and to combined stresses NaCl–SA and NaCl–JA for 10 days were obtained using SPAD-502Plus (Konica Minolta, Meudon, France). The measurement was repeated at least four times for each leaf of Wt plants and transformed *Arabidopsis* lines.

### 4.7. RNA Extraction and Gene Expression Profiles Analysis

In order to analyze the expression profiles of *PvPR10* genes in the common bean and a subset of stress-related genes in the Wt plants and the transformed *Arabidopsis* lines under salt and combined stress, the total RNA was extracted from leaves and stems of common beans exposed or not to different stress treatments, and from the aerial parts of Wt plants and the transformed Arabidopsis lines. The extraction was performed using the TRIzol reagent [84], and then each sample was quantified using a nanodrop.

To perform the synthesis of the first strand cDNA, firstly, the residual genomic DNA was eliminated using 1 U of RNase-free DNase (Thermo Fisher Scientific, ON, Canada), and the reaction was incubated for 10 min at 37 °C. Then, the first strand cDNA of each sample was synthesized using M-MLV reverse transcriptase (Invitrogen, CA, USA) and the Oligo-dT primer (18 mer). The qRT-PCR reactions were prepared as described by Feki et al. [6], using the CFX96 Real-Time PCR detection system (Bio-Rad, Ermont, France) and 0.5 μL of each primer (10 μM). The primers used in qRT-PCR reactions for the expression analysis of *PvPR10* genes in common beans were described previously [21], while those used for the expression analysis of the SA-, JA-, and ABA-related genes in the Wt and transgenic *Arabidopsis* plants are detailed in Supplementary Table S1. The two reference genes, *Arabidopsis* ubiquitin 10, *UBQ10* (AT4G05320), and *Phaseolus vulgaris* actin *Pvactin* (Phvul.008G011000) were used as internal control of qRT-PCR reactions. These primers are also described in Table S1. The results of qRT-PCR reactions were presented in heat maps that were drawn by means of TBtools-II software [85].

### 4.8. Quantification of Phenolic Compounds and ABA Levels

The phenolic compounds were extracted using 10 mg of the leaves of the Wt plants and the transformed *Arabidopsis* lines exposed or not to salt and to combined stresses NaCl–SA and NaCl–JA; dry matter was ground with 1 mL of ethanol 70% after incubation at 4 °C for 24 h. Then, each sample was filtered with a Whatman filter paper (no. 4). The total phenolic content was determined using Folin–Ciocalteu reagent and following the method of Dewanto et al. [86]. The total phenolic content was expressed as µg gallic acid equivalent per g dry weight (µg GAE g^−1^ DW). For ABA level quantification, the HPLC assay was performed using a reversed-phase C18 analytical column of 4.6 × 100 mm and 3.5 µm particle size, and the DAD detector was set to 235 nm [87].

### 4.9. Statistical Analysis

For statistical analysis, the statistical software SPSS ver.20 was used and based on the ANOVA method. Means were compared using Tukey’s HSD test. Different letters indicate significant differences (*p* < 0.05).

## 5. Conclusions

The present study demonstrates the crucial role of the common bean protein *PvPR10-3* in conferring salt stress tolerance in *Arabidopsis*. We showed that the *PvPR10-3* gene was up-regulated in common bean leaves and stems treated with NaCl and foliar application of JA. Heterologous expression of *PvPR10-3* in *Arabidopsis* enhanced tolerance to salt and phytohormone stress, an effect that was significantly amplified by exogenous JA. We determined that JA, in contrast to SA, potentiates the salt stress response in transgenic lines by stimulating ABA and phenolic compound accumulation and enhancing ROS scavenging through the activation of H_2_O_2_-detoxifying enzymes. While not directly measured in this study, the observed accumulation of ABA suggests that improved stomatal regulation may be a contributing factor to the maintained photosynthetic performance and water balance in the transgenic lines under salt stress. We propose a model for the molecular mechanism of the *PvPR10-3*-mediated tolerance to the combined NaCl–JA stress, which highlights the key components of the crosstalk between JA, ABA, and SA pathways. However, to better understand the mechanisms underlying this combined stress tolerance, further studies are required to identify additional components associated with the *PvPR10-3* protein and JA signaling.

## Data Availability

The data are contained within the article and the Supplementary Materials.

## Supplementary Materials

The following Supporting Information can be downloaded at: https://www.mdpi.com/article/10.3390/plants14193092/s1, Figure S1. (A) Schematic diagram of the T-DNA region of the binary vector pBI321. The ORF of *PvPR10-3* was cloned downstream of the constitutive cauliflower mosaic virus (CaMV) 35S promoter between the restriction sites X*ba*I and B*amH*I. The hygromycin resistance gene (HPTII) is expressed under the control of the nopaline synthase promoter (PNOS) and terminator (TNOS). (B) PCR analysis confirming the genomic integration of the *PvPR10-3* transgene in the six transgenic lines. (C) RT-PCR analysis confirming the expression of the *PvPR10-3* transgene in the six transgenic lines. Wild-type (Wt) plants showed no amplification of the transgene. M: Molecular weight. The *Arabidopsis AtActin* gene was used as an internal control. C (-) negative control without cDNA. Table S1. Sequences of primers used in *PvPR10-3* isolation, its cloning, and in qRT-PCR reactions. The underlined sequences correspond to the restriction sites of the enzymes X*ba*I and B*amH*I in the primers PR10-F and PR10-R, respectively.
