# Supplementary material for: PvPR10-3 Expression Confers Salt Stress Tolerance in Arabidopsis and Interferes with Jasmonic Acid and ABA Signaling
Source: Plants (Basel). 2025 Oct 7;14(19):3092. doi: 10.3390/plants14193092 (PMC12526191; doi:10.3390/plants14193092)
Supplement: Supplementary file 1 [file plants-14-03092-s001.zip › plants-3880511-supplementary.pdf]

plants

MDPI

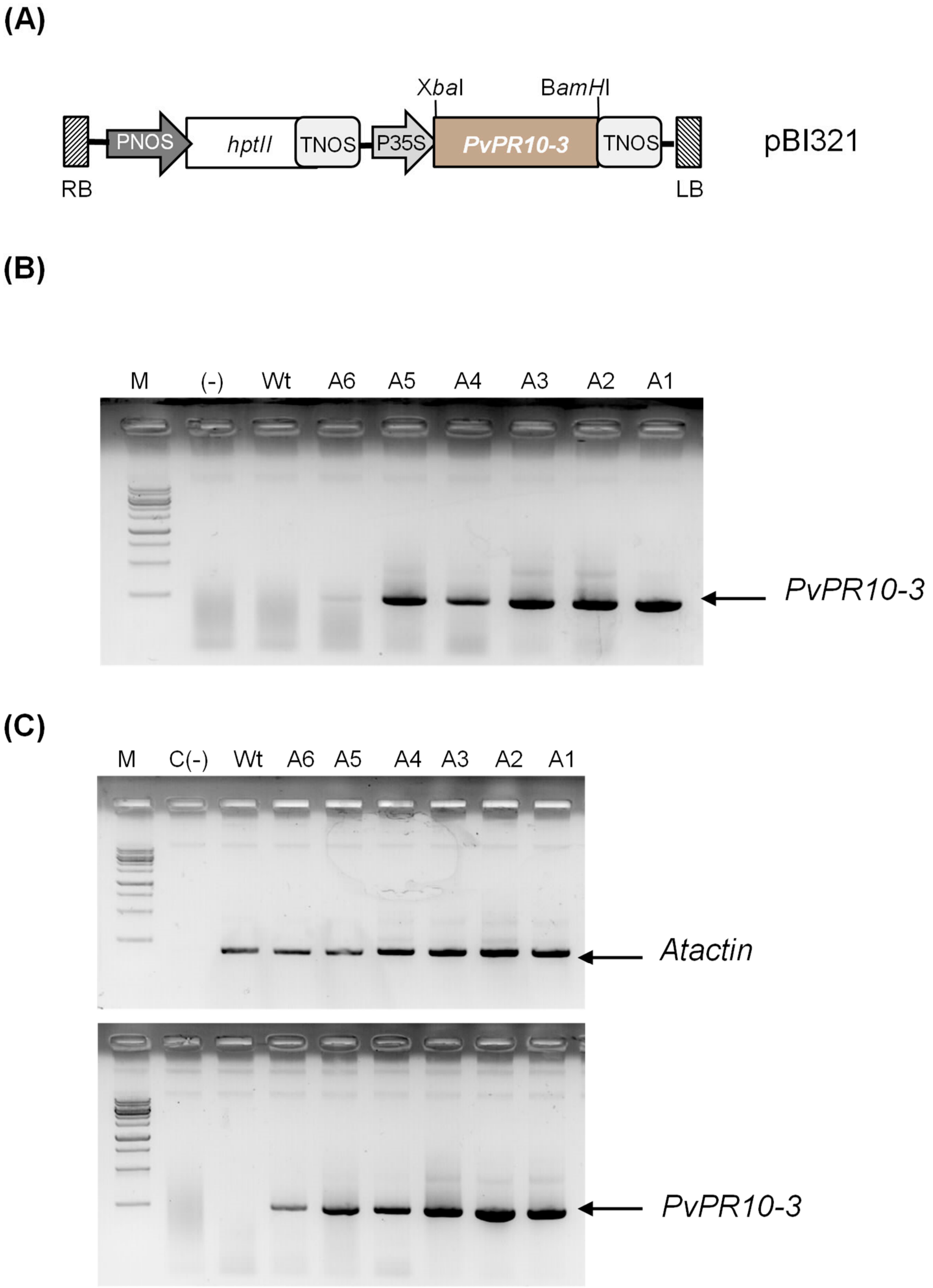

Figure S1. (A) Schematic diagram of the T-DNA region of the binary vector pBI321. The ORF of *PvPR10-3* was cloned downstream of the constitutive cauliflower mosaic virus (CaMV) 35S promoter between the restriction sites *Xba*I and *BamH*I. The hygromycin resistance gene (HPTII) is expressed under the control of the nopaline synthase promoter (PNOS) and terminator (TNOS). (B) PCR analysis confirming the genomic integration of the *PvPR10-3* transgene in the six transgenic lines. (C) RT-PCR analysis confirming the expression of the *PvPR10-3* transgene in the six transgenic lines. Wild-type (Wt) plants showed no amplification of the transgene. M: Molecular weight. The *Arabidopsis AtActin* gene was used as an internal control. C (-) negative control without cDNA.

Table S1. Sequences of primers used in *PvPR10-3* isolation, its cloning, and in qRT-PCR reactions. The underlined sequences correspond to the restriction sites of the enzymes *Xba*I and B*amH*I in the primers PR10-F and PR10-R, respectively.

Primer names	Primer sequences (5′-3′)	Description
PR10-F	ACTCTAGAATGGGTGTTTTCACATTCGAGGAT	Isolation and cloning of *PvPR10-3*
PR10-R	TCGGATCCTCAGGGATTGGCCAGAAGGTATG	
ActFA	GGC GAT GAA GCT CAA TCC AAA	*AtActin* (reference gene in *Arabidopsis*)
ActRA	GGT CAC GAC CAG CAA GAT CAA	
UBQ-F	TCAAGGCCAAGATCCAGGAC	*UBQ10* (AT4G05320) (reference gene in *Arabidopsis*)
UBQ-R	TGGATGTTGTAGTCGGCCAA	
ActinF-F	TGCATACGTTGGTGATGAGG	*Pvactin* (Phvul.008G011000) (reference gene in common bean)
ActinF-R	AGCCTTGGGGTTAAGAGGAG	
DREB-F	AACCTGTCAGCAACAACAGC	AtDREB2A (AT5GG05410)
DREB-R	AAACACATCGTCGCCATTTA	
ERD1-F	GTCAAGATGAGGCGGTAGC	AtERD1 (AT5G51070)
ERD1-R	GTCCACAGAAAAGCATAGCAG	
RD29A-F	AAGCAATGAGCATGAGCAAG	AtRD29A (AT5G52310)
RD29A-R	GGAAGACACGACAGGAAACAC	
RD22-F	GGTTCGGAAGAAGCGGAGAT	AtRD22 (AT5G25610)
RD22-R	AGTGGAAACAGCCCTGACGT	
GI-F	GTTTCTCGACACTGCGGC	GI (AT1G22770)
GI-R	ACGTCCAAATGTTTTGTCTAGAG	
FL-F	GAGTCGATAGGAGGAGGTAGC	FL (AT3G10390)
FL-R	TCGTCTCACCACCAACATGT	
FT-F	CGCCAGAACTTCAACACTCG	FT (AT1G65480)
FT-R	TTCTTCCTCCGCAGCCAC	
TFL1-F	TGTGTTTGTTCTGTTCAGGCA	TFL1 (AT5G03840)
TFL1-R	CCAAGATCATACTCGACCGC	
NIN-F	GTCACATACCGGTACAACGC	NINJA (AT4G28910)
NIN-R	GGCTAACGTACTCCTCGCTT	
JAZ1-F	AAGAGCTTCACTTCACCGG	JAZ1 (AT1G19180)
JAZ1-R	GCCTGTGGTTTGAGGGTTTG	
JAZ2-F	CGAACTCCCGATTGCAAGAA	JAZ2 (AT1G74950)
JAZ2-R	GGCTTGGAAGACGCTTCAG	
JAZ6-F	CAAGCCAGAGATGGTTGCTC	JAZ6 (AT1G72450)
JAZ6-R	CCTTCTTTCTTGTCCACCTCC	
JAZ11-F	AGGAGACGTTCACTTCAAAGATT	JAZ11 (AT3G43440)
JAZ11-R	ACAATGGGGCTGGTTTCATTTT	
JAZ12-F	ACGGCTGATCTACCTATTGCA	JAZ12 (AT5G20900)
JAZ12-R	AGTCTGAAGTAGGGTAAGGGT	
MYC2-F	GGCGTTGATGGATTTGGAGT	MYC2 (At1g32640)
MYC2-R	GCTGTTCTTGCGTATAGATCCT	
MYC3-F	AGGAGTTGGATTTGGAAGTGAA	MYC3 (At4g17880)
MYC3-R	CTTGAGTTGATCTTGCGTGAAA	
PR2-F	GGCCAGGGAAAGCTATAGAGA	PR2 (at3g57260)
PR2-R	ACTTAGACTGTCGATCTGGATGA	
TGA-F	GCCAGGCTGATAATTTGAGACTA	TGA2 (at5g06950)
TGA-R	GCCGTGAGAAGTAATCGTGT	
WR-F	GGTTTGCCTCACCCGTATATG	WRKY2 (at5g56270)

WR-R	TGTCCTGAATCTGAAACCGGT	
SID-F	GGGACAGGGATAGTAGCTGG	SID2 (at1g74710)
SID-R		
EDS-F	AATCGCCTGTAGAGATGTTGT	EDS1 (at3g48090)
EDS-R	AATGGATCACAGACGGGGAG	
PAD-F	GAGGAGAATGCGATTTGTGATT	PAD4 (at3g52430)
PAD-R	TTGGATGTTAAAGCGAAGAACTC	
ALD1-F	TGCGTCACTCTCATCAACAAC	ALD1 (at2g13810)
ALD1-R	CCCGGTGGAGAAGAGTATTTG	
	TGTTTGGTTCGTGTGTTAAAGAA	
